# Molecular connectivity studies in neurotransmission: a scoping review

**DOI:** 10.1162/imag_a_00530

**Published:** 2025-04-04

**Authors:** Mario Severino, Débora Elisa Peretti, Marjorie Bardiau, Carlo Cavaliere, Matthieu Doyen, Gabriel Gonzalez-Escamilla, Tatiana Horowitz, Martin Nørgaard, Jhony Alejandro Mejia Perez, Matej Perovnik, Michael Rullmann, Dilara Steenken, Daniel Talmasov, Chunmeng Tang, Tommaso Volpi, Zhilei Xu, Alessandra Bertoldo, Vince D. Calhoun, Silvia Paola Caminiti, Xin Di, Christian Habeck, Sharna Jamadar, Daniela Perani, Arianna Sala, Vesna Sossi, Igor Yakushev, Joana B. Pereira, Mattia Veronese

**Affiliations:** Department of Information Engineering, University of Padua, Padua, Italy; Laboratory of Neuroimaging and Innovative Molecular Tracers, University of Geneva, Geneva, Switzerland; ULiège Library, University of Liège, Liège, Belgium; IRCCS SYNLAB SDN, Naples, Italy; Université de Lorraine, IADI, INSERM U1254, Nancy, France; Department of Neurology, Focus Program Translational Neuroscience, Rhine-Main, Neuroscience Network, University Medical Centre of the Johannes Gutenberg University Mainz, Mainz, Germany; Department of Nuclear Medicine, Timone Hospital, AP-HM, Aix Marseille Univ, CERIMED, CNRS, Centrale Marseille, Institut Fresnel, Marseille, France; Department of Computer Science, University of Copenhagen, Copenhagen, Denmark; Molecular Imaging Branch, National Institute of Mental Health, Bethesda, MD, United States; Memory and Aging Centre, Department of Neurology, University of California San Francisco, San Francisco, CA, United States; Department of Neurology, University Medical Centre Ljubljana, Ljubljana, Slovenia; Department of Nuclear Medicine, University of Leipzig, Leipzig, Germany; Department of Nuclear Medicine, TUM School of Medicine and Health, Munich, Germany; Departments of Neurology and Psychiatry, Columbia University Irving Medical Centre, New York, NY, United states; Nuclear Medicine and Molecular Imaging, Department of Imaging and Pathology, KU Leuven, Leuven, Belgium; Department of Radiology and Biomedical Imaging, PET Centre, Yale University, New Haven, CT, United States; Division of Neuro, Department of Clinical Neuroscience, Karolinska Institutet, Stockholm, Sweden; Padova Neuroscience Centre, University of Padua, Padua, Italy; Tri-Institutional Centre for Translational Research in Neuroimaging and Data Science (TRenDS), Georgia State, Georgia Tech, Emory, Atlanta, GA, United States; Department of Brain and Behavioural Sciences, University of Pavia, Pavia, Italy; Department of Biomedical Engineering, New Jersey Institute of Technology, Newark, NJ, United states; Cognitive Neuroscience Division, Department of Neurology, Columbia University Irving Medical Centre, New York, NY, United States; School of Psychological Sciences, Monash University, Melbourne, Victoria, Australia; Monash Biomedical Imaging, Monash University, Melbourne, Victoria, Australia; Vita-Salute San Raffaele University, Milan, Italy; Coma Science Group, GIGAConsciousness, University of Liege, Liege, Belgium; Centre du Cerveau2, University Hospital of Liege, Liege, Belgium; Department of Physics and Astronomy, University of British Columbia, Vancouver, BC, Canada; Department of Neuroimaging, King’s College London, London, United Kingdom

**Keywords:** molecular connectivity, molecular imaging, neuroimaging, neurotransmission, positron emission tomography

## Abstract

Positron emission tomography (PET) and single photon emission computed tomography (SPECT) are essential molecular imaging tools for the in vivo investigation of neurotransmission. Traditionally, PET and SPECT images are analysed in a univariate manner, testing for changes in radiotracer binding in regions or voxels of interest independently of each other. Over the past decade, there has been an increasing interest in the so-called*molecular connectivity*approach that captures relationships of molecular imaging measures in different brain regions. Targeting these inter-regional interactions within a neuroreceptor system may allow to better understand complex brain functions. In this article, we provide a comprehensive review of molecular connectivity studies in the field of neurotransmission. We examine the expanding use of molecular connectivity approaches, highlighting their applications, advantages over traditional methods, and contributions to advancing neuroscientific knowledge. A systematic search in three bibliographic databases MEDLINE, EMBASE, and Scopus on July 14, 2023 was conducted. A second search was rerun on April 4, 2024. Molecular imaging studies examining functional interactions across brain regions were included based on predefined inclusion and exclusion criteria. Thirty-nine studies were included in the scoping review. Studies were categorised based on the primary neurotransmitter system being targeted: dopamine, serotonin, opioid, muscarinic, glutamate, and synaptic density. The most investigated system was the dopaminergic and the most investigated disease was Parkinson’s disease (PD). This review highlighted the diverse applications and methodologies in molecular connectivity research, particularly for neurodegenerative diseases and psychiatric disorders. Molecular connectivity research offers significant advantages over traditional methods, providing deeper insights into brain function and disease mechanisms. As the field continues to evolve, embracing these advanced methodologies will be essential to understand the complexities of the human brain and improve the robustness and applicability of research findings in clinical settings.

## 1 Introduction

### 1.1 PET and SPECT to study neurotransmission

**Fig. 1:**
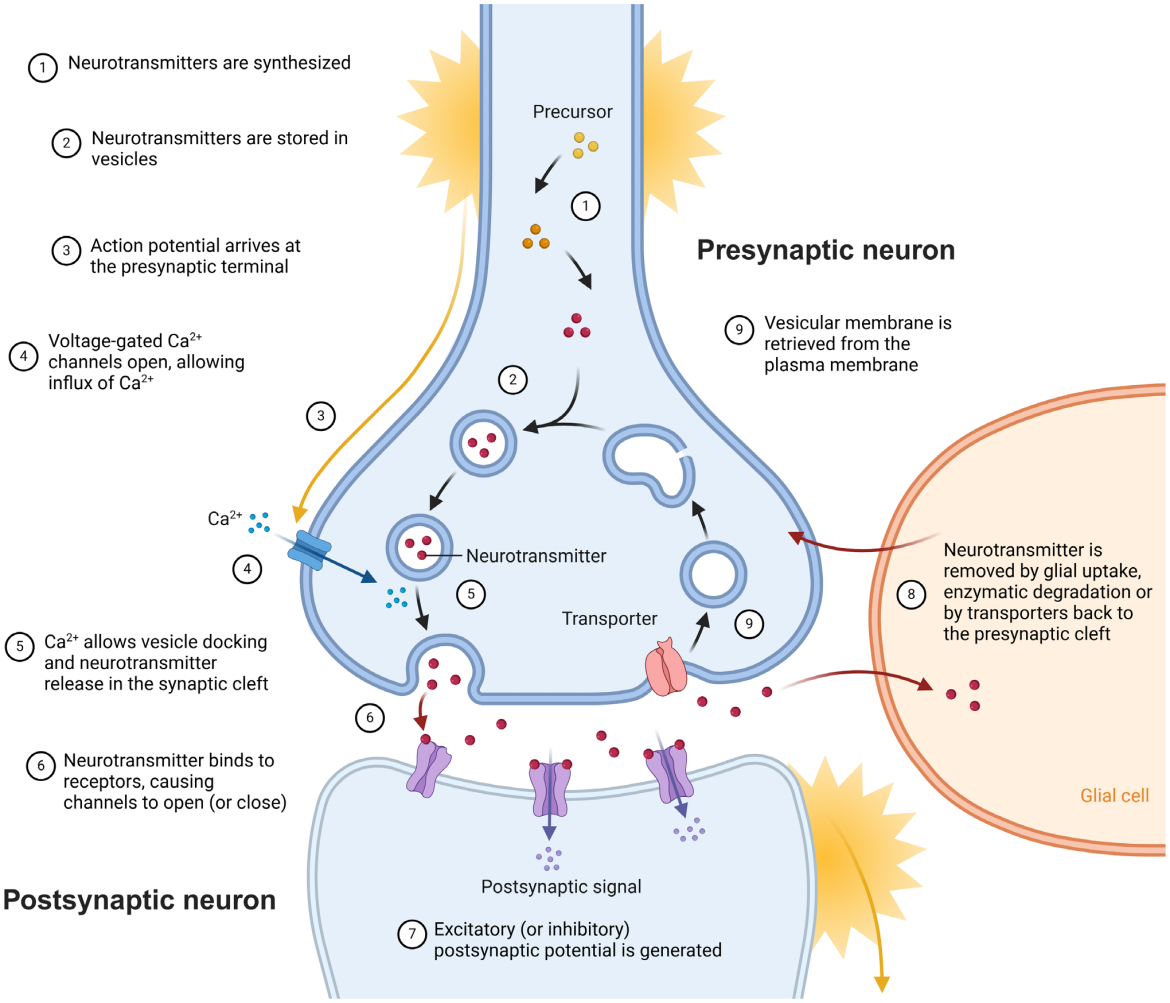
Neurotransmission: 1) Neurotransmitters are synthesised. 2) Neurotransmitters are stored in vesicles. 3) Action potential arrives at the presynaptic terminal. 4) The action potential causes the opening of voltage-gated Ca²^+^channels allowing the influx of calcium ions. 5) Ca²^+^allows vesicles docking and neurotransmitters release in the synaptic cleft. 6) Neurotransmitter binds to receptor causing the opening or closing of channels. 7) Postsynaptic potential is generated. 8) Neurotransmitters are removed back to the presynaptic cleft. 9) Vesicular membranes are retrieved from the plasma membrane (Figure created inhttps://www.biorender.com/).

Neurotransmission is the primary process by which neurons communicate and represents a biological pillar to all functions of the central and peripheral nervous system, including sensation, movement, cognition, and, ultimately, individual behaviour (Jessell & Kandel, 1993). Neural communication can occur through two main modalities of synaptic transmission: chemical and electrical. At chemical synapses, information is transferred via the release of neurotransmitters from one cell, which are detected by an adjacent cell (Südhof & Malenka, 2008). In contrast, at electrical synapses, the cytoplasm of adjacent cells is directly connected by clusters of intercellular channels called gap junctions (Bennett & Zukin, 2004). Chemical synapses are more common in the human brain (Pereda, 2014). Neurotransmission at these synapses depends on the interaction between molecular and electrical signals (Fig. 1), starting with the action potential, an electrical signal generated in the neuron’s cell body, which travels down the axon towards the synapse. When the action potential reaches the axon terminal or presynaptic terminal, it triggers the opening of voltage-gated calcium channels. Calcium ions (Ca²^+^) enter the presynaptic terminal through these channels, causing synaptic vesicles to fuse with the presynaptic membrane. As a result, the neurotransmitters contained in the vesicles are released into the synaptic cleft—the space between the presynaptic and postsynaptic neurons—where they transmit the signal by binding to the receptors of the postsynaptic neuron. This binding activates or inhibits the postsynaptic neuron, affecting numerous other neurons within specific pathways that are essential for maintaining the homeostatic balance of neuronal activity and overall healthy brain function (Gunn et al., 2015;Sudhof, 2012).

Dysregulation and alterations of neurotransmitter and neuroreceptor levels and functions, whether due to deficiency or excess, are implicated in the pathophysiology of numerous neurodegenerative conditions such as Alzheimer’s disease (AD) and Parkinson’s disease (PD) (Warren et al., 2017), as well as psychiatric disorders like Schizophrenia and Major Depressive Disorder (MDD) (Nimgampalle et al., 2023;Sonnenschein et al., 2020). Therefore, elucidating the mechanisms of neurotransmission in vivo is paramount for advancing our understanding of brain function in both health and disease states and for the development of novel pharmacological treatment.

**Fig. 2:**
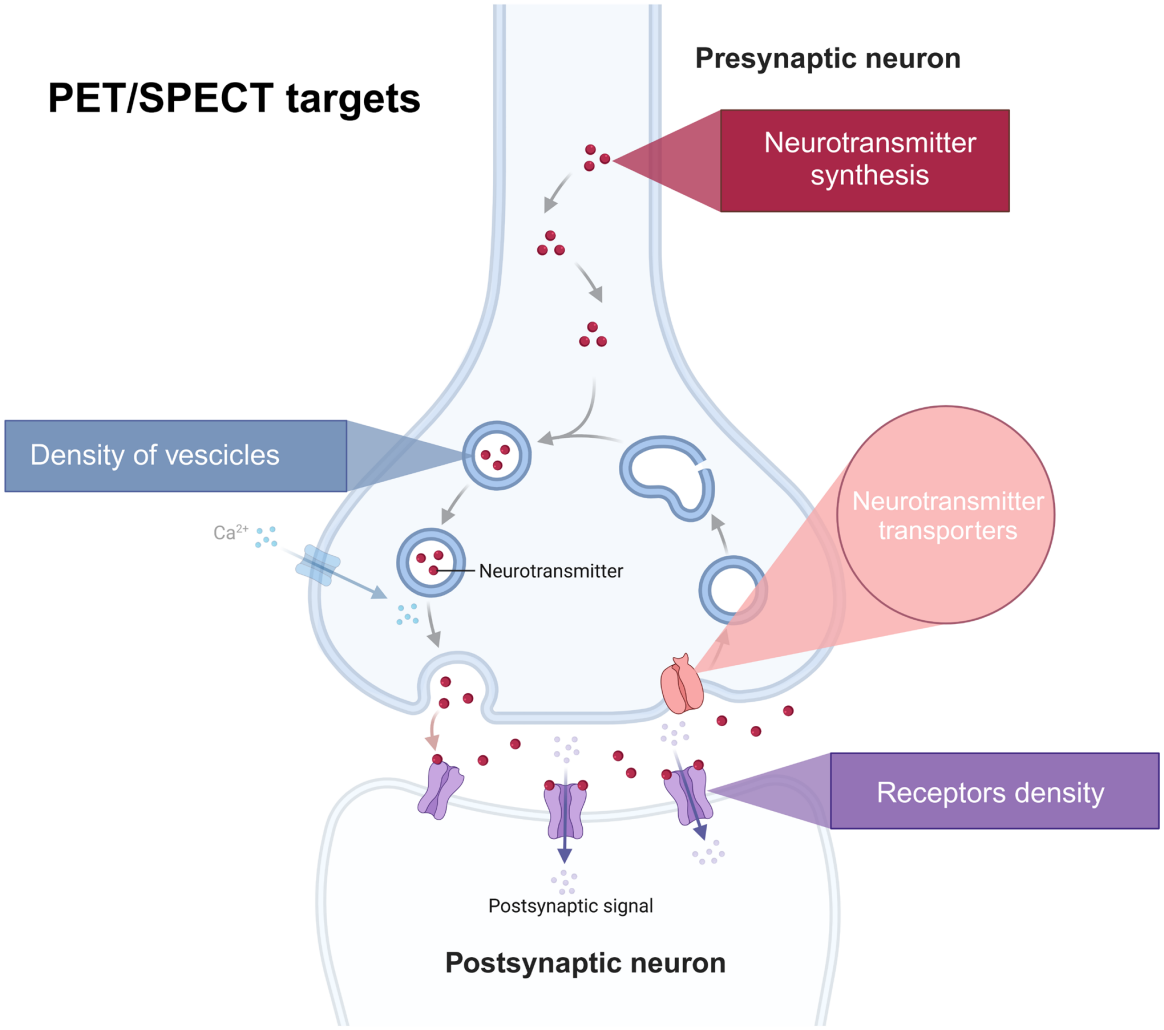
Targets of neurotransmission measurable with PET and SPECT imaging (Figure created inhttps://www.biorender.com/).

Neurotransmission can be studied in vivo using molecular imaging tools, including Positron Emission Tomography (PET) and Single Photon Emission Computed Tomography (SPECT) (Kimura et al., 2020). These imaging modalities are powerful tools for measuring the local concentration of diverse molecular targets with remarkable sensitivity and specificity in a non-invasive manner (Ametamey et al., 2008). Molecular imaging can visualise different aspects of neurotransmission (Fig. 2). According to the type of radiotracer used, it is possible to quantify neurotransmitter synthesis (e.g., dopamine synthesis (Volkow et al., 1996)), the concentration of synaptic vesicle density (e.g., SV2A (Toyonaga et al., 2019)), specific neurotransmitter types (such as the vesicular monoamine transporter 2 (VMAT_2_) (Carson et al., 2022)), the density and distribution receptors (e.g., D_2_/D_3_receptors for the dopamine system (Sokoloff & Le Foll, 2017;Xu et al., 2013), or 5HT_1A_/5HT_2A_receptors for the serotonin system (Kumar & Mann, 2015)), or neurotransmission transporters (e.g., DAT for dopamine (Varrone & Halldin, 2010) and SERT for the serotonin system (J. S. Kim et al., 2006). Finally, molecular imaging can be used to measure endogenous neurotransmitter levels and their release by detecting the competitive binding between endogenous neurotransmitters and radioligands to the same neuroreceptor sites (Giovacchini et al., 2005).

In medicine, molecular imaging has repeatedly proved to be an invaluable tool for probing neurotransmission alterations underlying many brain disorders and describing the spatiotemporal evolution of neurotransmission abnormalities throughout different stages of these diseases (Zhu et al., 2014). Some of the neurotransmission imaging methods have also translated into clinical work-up as diagnostic and monitoring biomarkers recommended by international clinical guidelines (Bega et al., 2021;Politis, 2014;Stoessl, 2012). The most well-known example is^123^I-FP-CIT SPECT imaging, used to identify and stage the degeneration of dopaminergic neurons in PD (Darcourt et al., 2010;Morbelli et al., 2020).

Finally, molecular imaging permits the temporal modelling of disease-related neurotransmission alterations, facilitating the evaluation of disease progression and the assessment of therapeutic interventions (Strafella et al., 2017).

### 1.2 Univariate versus network and multivariate approaches

Over the past two decades, the concept of the brain as a network has become central to neuroscience. This network-based perspective emphasises that brain functions emerge from the interaction of distributed regions within large-scale networks (Wig, 2017). While blood oxygenation level-dependent (BOLD) functional MRI (fMRI) has become the most widely used tool to study functional connectivity, due to its accessibility, cost-effectiveness, and lack of ionising radiation, it relies heavily on hemodynamic signals. Thus, this method alone cannot fully capture the complexity of brain activity, which involves both biochemical and electrical processes. In contrast, molecular imaging, which uses radiotracers to detect molecular targets with high sensitivity and specificity (Hahn et al., 2019), offers more accurate biological insights that complement fMRI. Given the limitations of single-modality approaches, there is a growing recognition of the need for an integrative, multimodal framework to comprehensively understand the brain’s connectome. Molecular imaging, with its unique capability to probe biochemical pathways, can play a crucial role in this integrative approach.

Nonetheless, traditional analysis methods in brain PET and SPECT research mainly focus on quantifying absolute tracer binding or uptake within specific brain regions (Gunn et al., 2001). While these approaches provide valuable insights into*in vivo*brain structure and activity, they possess limitations that warrant consideration. Region-wise analyses rely on a priori definition of anatomical or functional areas of the brain, potentially overlooking subtle or distributed effects across the brain (Gentili et al., 2021). Parametric voxel-wise analyses, on the other hand, depend on the image resolution of the scanner, reconstruction methods, partial volume correction (PVC), and reference regions used for normalisation. Moreover, regardless of the spatial resolution of the analysis, each volume of interest is typically treated as independent, ignoring brain spatial covariance. This often necessitates strict multiple comparison corrections to prevent inflated Type I error rates, which can result in overcorrection and increased Type II errors (Scarpazza et al., 2015). These limitations, alongside the spread of network science into neuroimaging field, motivated the development and validation of complementary multivariate and network methodologies in molecular neuroimaging, able to capture the complex interactions among brain regions and provide a more comprehensive understanding of brain functioning and disease pathology.

Multivariate methods are specifically used to address the complexities and interdependencies of neuroimaging data by simultaneously considering the interrelationships among multiple sources of information and possibly multiple volumes of interest (Chen et al., 2014). These methods facilitate the identification of intricate patterns of brain activity and structure that remain undetectable when analysing individual variables in isolation. For example, rather than examining the activity of a single brain region, multivariate methods allow for assessing the combined activity of several regions to determine their collective contribution to a specific task or condition (Clark & Stoessl, 1986). These methods can also identify combinations of brain activities that correspond to distinct cognitive states or differentiate between healthy and diseased brains, and between different diseases and stages. Furthermore, multivariate techniques can be employed to reduce the dimensionality of the data, extract a smaller set of key features, or integrate data from diverse sources (Habeck, 2010).

Parallel with multivariate approaches, network-based approaches, usually constructed using pair-wise correlation between regions or voxels, have been largely employed to study brain connectivity. A common feature of these network methods is the ability to construct a mathematical representation of the brain in the form of an adjacency matrix (Fornito et al., 2016). In this representation, brain regions are modelled as nodes, while the edges between them represent the biological or statistical interactions between these regions (Bullmore & Sporns, 2009;Fornito & Bullmore, 2015).

Although most of the recent multivariate and network approach findings in neuroimaging are derived from structural and fMRI studies, in recent years these methodological advances have increasingly been applied to PET/SPECT data (Yakushev et al., 2017). This paradigm shift follows a decade of evidence suggesting that neurotransmission and molecular pathological alterations underlying brain diseases invariably pass through large-scale brain networks (Goulas et al., 2021;Seeley et al., 2009). In this context,*molecular connectivity*refers to an approach that leverages molecular imaging to explore brain connectivity. This umbrella term is commonly used in the literature to describe the statistical interdependencies between regional measurements obtained from molecular imaging techniques (Sala et al., 2023). In the past few years, the term ‘molecular connectivity’ has been used to describe various methodologies aimed at constructing maps or matrices that reflect the statistical relationships between brain regions based on their molecular properties (as derived from PET or SPECT). These maps are generated through different statistical analyses, depending on the type of modality, tracer used, and the computational method chosen. Consequently, the biological interpretation and insights derived from the results can vary (Sala et al., 2022). One example is the computation of covariance matrices of regional PET signals across subjects. Up to today, this represents the most common approach used as a proxy for molecular connectivity. This method is favoured for its simplicity and the fact that it can be applied to static PET data, offering a broader perspective on shared connectivity patterns across populations (Sala & Perani, 2019). However, the limitation of estimating connectivity at the group level, rather than at the individual level, poses challenges for biological interpretation (Sala et al., 2022;Volpi et al., 2023). An alternative approach involves using dynamic data to construct molecular connectivity maps at the individual level. This method leverages temporal information from the radiotracer kinetics to compute connectivity through various computational techniques (Jamadar et al., 2021).

Other approaches to study brain connectivity that do not rely on the construction of an adjacency matrix are the scaled subprofile model (SSM), a multivariate principal component analysis (PCA)-based algorithm applied directly to voxel-by-voxel covariance data. In this case, an entire group image set can be reduced to a few significant linearly independent covariance patterns and corresponding subject scores (Spetsieris et al., 2013). Ultimately, another source-based multivariate method is independent component analysis (ICA), a data-driven computational procedure that decomposes or ‘un-mixes’ a measured signal into its maximal spatially independent ‘sources’ (Calhoun et al., 2009).

These approaches allow researchers to simultaneously explore variations in the relationships between multiple brain regions or patterns of activation, offering valuable insights into covarying patterns of tracer binding across the entire brain. From a physiological perspective, the interpretation of molecular connectivity for tracers targeting neurotransmission strictly depends on the specific neurotransmission system and target being studied. As the literature still lacks a mechanistic understanding to support signal changes in molecular-based networks, multiple answers can be proposed. For example, decreased connectivity might indicate selective denervation from neurotransmitter nuclei projecting to the target regions under evaluation (Sala & Perani, 2019). Meanwhile, both increased and decreased connectivity may reflect compensatory processes, as proposed byCaminiti et al. (2023),DuBois et al. (2021),Luo et al. (2023), andMihaescu et al. (2021).

### 1.3 Purpose of the study

This paper provides a comprehensive, state-of-the-art overview of brain connectivity analysis in the study of neurotransmission using molecular neuroimaging, presented through a scoping review. We examine the expanding use of molecular connectivity approaches, highlighting their applications, advantages over traditional methods, and contributions to advancing neuroscientific knowledge. Through an in-depth review, we provide researchers and clinicians with a clear understanding of the current landscape, highlighting key successes while outlining challenges and potential strategies to address them, with the goal of advancing future research and translating these approaches into clinical applications.

## 2 Materials and Methods

The PRISMA statement (Preferred Reporting Items for Systematic Reviews and Meta-analyses, guidelines extension for scoping reviews (PRISMA-ScR) was adhered to in conducting this scoping review (Tricco et al., 2018). The PRISMA-ScR checklist was used to perform the analysis. A study protocol was prepared in OSF prior to the initiation of data collection to ensure methodological rigour and transparency.

### 2.1 Search strategy

Original articles were searched for in three bibliographic databases (MEDLINE (via Ovid), EMBASE (via Elsevier), and Scopus (via Elsevier) on July 14, 2023. A second search was rerun on April 4, 2024. The search strategy consisted of two key concepts: (1) Positron Emission Tomography (PET) and Single Photon Emission Computed Tomography (SPECT) and (2) connectivity. The complete search strategy is listed inAnnex 1.

### 2.2 Eligibility criteria

Molecular imaging studies examining functional interactions across brain regions were included based on predefined inclusion and exclusion criteria. The eligibility criteria (inclusion and exclusion criteria) were defined as explained below. These criteria encompassed original studies that: a) employed brain PET or SPECT as imaging modalities, b) measured parameters such as blood flow, metabolism, neuroreceptor systems, protein/molecule synthesis, or protein/molecule density. Exclusion criteria comprised preclinical investigations, studies focusing on regions outside the brain, post-mortem analyses, animal studies, and those utilising monoclonal antibody imaging techniques. Finally, letters, commentaries, review papers, and conference abstracts were also excluded.Table 1summarises the inclusion and exclusion criteria used in this study.

**Table 1.** Eligibility criteria (inclusion and exclusion criteria) of references to be included in the scoping review.

	Inclusion criteria	Exclusion criteria
**Population**	Humans	Animals
**Concept**	Connectivity, covariance, network	
**Context**	PET and SPECT Measure: blood flow, metabolism, neuroreceptor systems, protein/molecule synthesis, or protein/molecule density	Regions outside the brain Post-mortem analyses Monoclonal antibody imaging techniques Preclinical investigations
**Sources**	Peer-reviewed original studies	Short commentaries Conference abstracts Reviews Letters to editors

### 2.3 Studies selection and data extraction

First, titles and abstracts were screened independently by two authors (DEP and MS) to exclude irrelevant records based on the eligibility criteria. A third author (MV) played the role of the third peer to arbitrate in case of disagreements. Then, the full text of each selected article was independently screened by 2 authors (DEP and MS). Additional studies were included a posteriori at the authors’ discretion. Notably, we included studies published prior to 1993 (formal definition of brain functional connectivity (Friston et al., 1993)), where the terminology deviates from connectivity, networks, or connectomics (term introduced in 2005 bySporns et al. (2005)). These studies were deemed relevant as they shared the overarching objective of investigating structural and functional interactions across brain regions. This inclusive approach ensured a comprehensive review of relevant literature, capturing both contemporary studies and earlier works contributing to the understanding of brain multi-scale architecture. Comprehensive information was extracted and organised into a pre-defined data sheet developed by the authors for each of these final articles. This information encompassed various aspects, including the names of the authors, year of publication, characteristics of the study population (both healthy control and patient groups), PET/SPECT tracer utilised, putative marker type and specification, protocol and analysis type, methods employed for connectivity analysis, software utilised, main findings, validation type (if applicable), multimodality type (if applicable), and any reported measures of multimodality similarity and performance. This systematic extraction process ensured thorough documentation of relevant details from each included study, facilitating comprehensive analysis and synthesis of findings.

## 3 Results

### 3.1 Search results

**Fig. 3:**
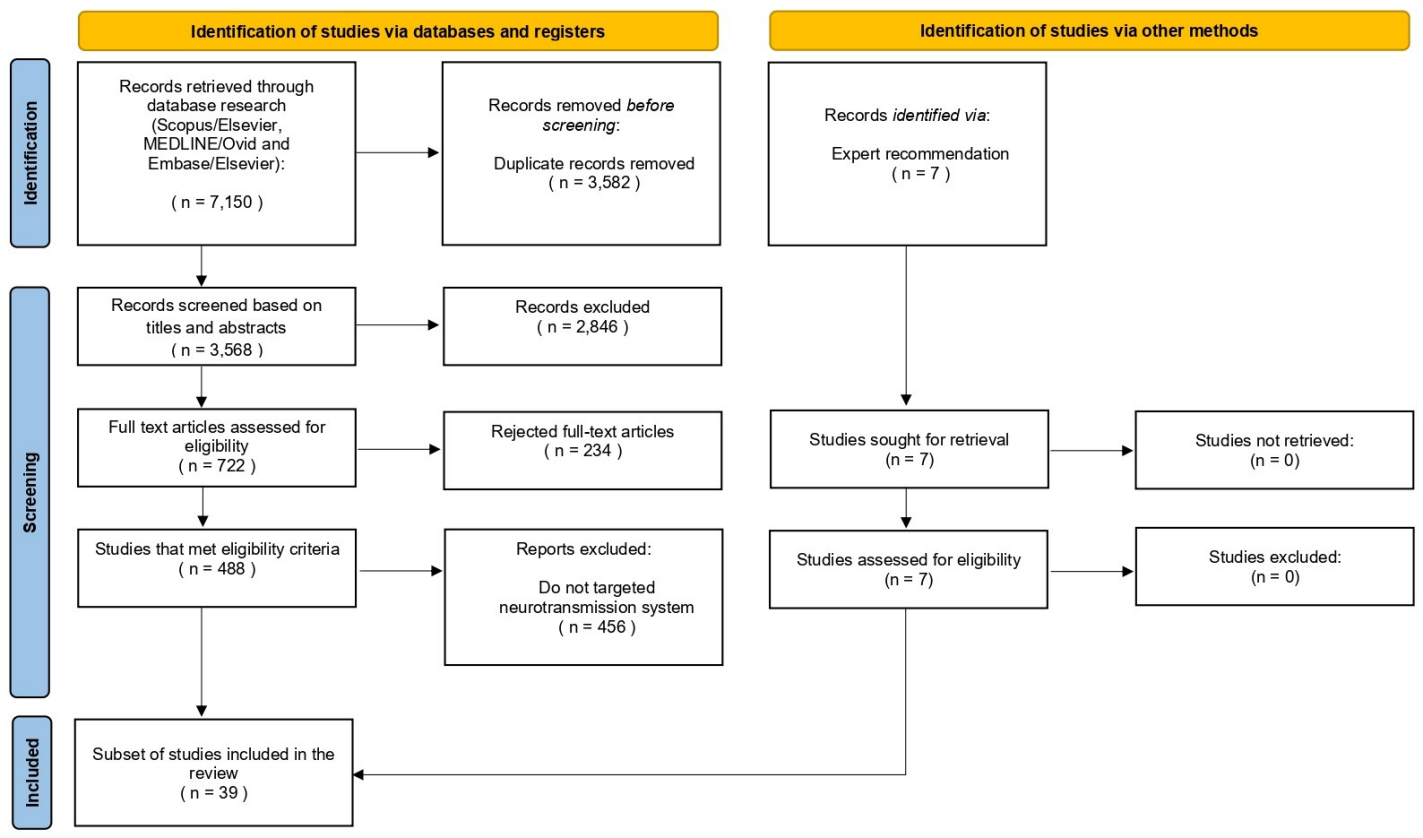
PRISMA flowchart for studies ‘selection.

After removing duplicates, a total of 3,568 references were retrieved from database searches (3,213 in July 2023 and 355 in April 2024). Following title and abstract screening, 722 references were selected for full-text review. Ultimately, 488 of these met the eligibility criteria and underwent data extraction. Full texts were excluded primarily due to incorrect study type or unsuitable analysis (i.e., lacking molecular connectivity). From the eligible studies, a subset of 32 articles specifically addressing neurotransmission systems was included in this review. Additionally, 7 more studies recommended by experts were screened and added to the scoping review, resulting in a final total of 39 articles. The identification of these articles was based mainly on the molecular probe used and its main binding targets.Fig. 3shows the PRISMA flow chart describing the articles’ selection process.

### 3.2 Studies characteristics

Studies were categorised based on the primary neurotransmitter system being targeted (Table 2). In instances where a study investigated multiple neurotransmitters, it was assigned to a single category based on the predominant target. The resulting sample comprised 23 studies targeting the dopamine system (radiotracers used:^11^C-FLB457,^18^F-FDOPA,^11^C-FeCIT,^18^F-Fallypride,^11^C-MP,^11^C-FLB457,^11^C-Raclopride,^18^F-FEOBV,^18^F-CFT,^11^C-CFT,^11^C-SCH23390,^18^F-FPCIT,^123^I-FP-CIT,^11^C-DTBZ,^11^C(+)-PHNO), 9 studies focusing on the serotonin system (radiotracers used:^11^C-SB217045,^11^C-WAY-100635,^11^C-DASB,^11^C-MADAM), 2 studies examining µ-opioid receptors (radiotracer used:^11^C-Carfentanil), 2 studies assessing synaptic density (radiotracer used:^11^C-UCB-J), 2 studies targeting muscarinic receptors (radiotracer used:^123^I-QNB), and 1 study investigating glutamate receptors (radiotracer used:^11^C-ABP688). The most frequently utilised outcome measure across these studies was the tracer binding potential (*BP*_ND_) (Mintun et al., 1984) employed by 20 studies. This metric represents the equilibrium ratio of the concentration of specifically bound radioligand to the combined concentration of free and non-specifically bound radioligand. Other studies looked at additional parameters as proxies for tracer-specific activity, such as, Specific Binding Ratio (*SBR*), used in 4 studies, Standardized Uptake Value ratio (*SUV*_r_), also used in 4 studies, the distribution volume ratio (*DVR*), in 3 studies, and total distribution volume (*V*_T_), used in 2 studies.

**Table 2.** Table summarising the findings of the reviewed studies.

Authors	Major findings	PET tracer	Putative marker	Population	METHOD
Yasuno et al. (2005)	A systems-level change, reflected in the connectivity of regional D _2_ receptor binding, was observed in individuals with schizophrenia.	^11^ C-FLB457	Dopaminergic D _2_ Receptors	HC (n = 19) and Schizophrenia (n = 10)	Inter-regional correlation
Kaasinen et al. (2006)	PCA revealed a specific component related to the thalamus and cerebellum in ^18^ F-FDG uptake, associated with both ^18^ F-FDOPA uptake and disease severity, which was not identified using univariate analysis.	^18^ F-FDG and ^18^ F-FDOPA	Glucose Metabolism and Dopa-Decarboxylase	PD (n = 25)	Inter-regional correlation
Wager et al. (2007)	Interregional correlations suggested a mechanism for placebo-induced pain relief mediated by the endogenous opioid system.	^11^ C-Carfentanil	µ-Opioid Receptors	HC (n = 15)	Pairwise correlation
Hahn et al. (2010)	Enhanced correlation between autoreceptor and heteroreceptor binding was observed after escitalopram treatment.	^11^ C-WAY-100635	Serotonin 5HT _1A_ Receptors	HC (n = 36) and Anxiety (n = 21)	Inter-regional correlation
Cervenka et al. (2010)	Striatal dopaminergic biomarkers may not reliably index global dopamine function.	^11^ C-FLB457 and ^11^ C-raclopride	Dopaminergic D _2_ Receptors	HC (n = 16)	Inter-regional correlation
Tuominen et al. (2014)	Regionally specific intercorrelations between opioid and serotonin tracers were identified in areas associated with several neuropsychiatric disorders.	^11^ C-MADAM and ^11^ C-Carfentanil	Serotonin Transporter and µ-Opioid Receptors	HC (n = 21)	Inter-regional correlation
Hahn et al. (2014)	A disturbance in a major 5-HT pathway in MDD was identified using an interregional correlation approach, complementing the understanding of the disease’s biological mechanisms.	^11^ C-DASB	Serotonin Transporter	HC (n = 20) and MDD (n = 20)	Inter-regional correlation
Nørgaard et al. (2017)	A whole-brain pattern of 5-HTT levels was identified that distinguished females without SAD from those with SAD.	^11^ C-DASB	Serotonin Transporter	Non-SAD (n = 13) and SAD (n = 6)	Multivariate PLS
Vanicek et al. (2017)	Significant interregional differences in SERT *BP*_ND_ correlations were observed between adult patients with ADHD and healthy controls.	^11^ C-DASB	Serotonin Transporter	HC (n = 25) and ADHD (n = 25)	Pairwise correlation
Worhunsky et al. (2017)	Cocaine-related alterations in D _2_ and D _3_ may extend beyond the dorsal striatum and midbrain to include the pallidum and ventral striatum.	^11^ C-(+)-PHNO	Dopaminergic D _2_ and D _3_ Receptors	HC (n = 26) and CUD (n = 26)	ICA
Caminiti et al. (2017)	Findings indicated greater presynaptic degeneration in the dorsal putamen than in the substantia nigra, with more severe molecular connectivity alterations in the nigrostriatal pathway compared to the mesolimbic pathway in PD.	^11^ C-FeCIT	Dopamine Transporter	HC (n = 14) and PD (n = 26)	Pairwise correlation
J. Kim et al. (2018)	Spatial organisation of D _2_ and D _3_ receptor availability, along with related functional connectivity, was significantly disrupted in stable outpatients with schizophrenia on antipsychotics.	^18^ F-Fallypride	Dopaminergic D _2_ Receptors	HC (n = 14) and Schizophrenia (n = 11)	Pairwise correlation
Klyuzhin et al. (2018)	Predominant voxel-level binding patterns in PD subjects were associated with the initial symptom onset and disease progression.	^11^ C-DTBZ and ^11^ C-raclopride	Dopamine Transporter	HC (n = 10) and PD (n = 41)	PCA - LASSO
Fu et al. (2018)	A serotonergic spatial covariance pattern characteristic of PD was identified, strongly correlated with disease duration and dopaminergic denervation measured using ^11^ C-DTBZ PET imaging.	^11^ C-DASB	Serotonin Transporter	HC (n = 9) and PD (n = 30)	SSM-PCA
Pillai et al. (2019)	Compromised structural and compensatory mechanisms of post-synaptic receptor regulation were observed in men with MDD.	^11^ C-WAY-100635	Serotonin 5HT _1A_ Receptors	HC (n = 20) and MDD (n = 16)	Inter-regional correlation
Ashok et al. (2019)	A significant global increase in µ-opioid receptor connection strength was observed in schizophrenia patients compared to controls, highlighting aberrant µ-opioid system activity.	^11^ C-Carfentanil	µ-opioid Receptors	HC (n = 20) and Schizophrenia (n = 19)	Pairwise correlation
Fu et al. (2019)	A method was introduced that captures the spatial and temporal disease patterns of PD, proving more sensitive to disease discrimination and progression compared to univariate analysis.	^18^ F-DBTZ and ^11^ C-MP	Dopamine Transporter and VMAT _2_	PD (n = 15)	Canonical correlation analysis
Veronese et al. (2019)	Graph metrics were shown to complement standard PET analysis, helping to understand how biological functions are organised across brain regions in both healthy and pathological conditions.	^18^ F-FDG, ^18^ F-FDOPA and ^11^ C-SB217045	Glucose Metabolism, Dopa-Decarboxylase and Serotonin 5HT _4_ Receptors	HC (n = 80), AD (n = 76) and MCI (n = 137)	Pairwise correlation
Verger et al. (2020)	Advantages of using ^18^ F-FDOPA PET imaging for molecular connectivity include its superior sensitivity and specificity compared to ^18^ F-FDG metabolic connectivity, particularly in revealing the mesotelencephalic system.	^18^ F-FDG and ^18^ F-FDOPA	Glucose Metabolism and Dopa-Decarboxylase	HC (n = 47)	Inter-regional correlation
Fazio et al. (2020)	The serotonergic system may become increasingly involved in PD patients as the disease progresses.	^11^ C-MADAM	Serotonin Transporter	HC (n = 20) and PD (n = 20)	Pairwise correlation
Colloby et al. (2020)	Baseline spatial covariance patterns of M1/M4 receptors were identified, distinguishing DLB from healthy individuals and associated with positive changes in global cognition.	^123^ I-QNB and 99mTc-exametyzime	Muscarinic Receptors M1 and M4 and Cerebral blood flow	Elderly controls (n = 24) and DLB (n = 14)	PCA spatial covariance
Smart et al. (2020)	Assessed the utility of four-dimensional ICA in a competition binding PET study, successfully separating each receptor subtype component without any a priori assumptions.	^11^ C-(+) PHNO	Dopaminergic D _2_ and D _3_ Receptors	HC (n = 8)	ICA
Mihaescu et al. (2021)	Connectivity dysregulation in extrastriatal dopamine networks may contribute to cognitive decline.	^11^ C-FLB457	Dopaminergic D _2_ Receptors	HC (n = 13), MCI (n = 17) and PD (n = 13)	Pairwise correlation
Sala et al. (2021)	Provided biological in vivo evidence for a significant disruption of the meso-limbic dopaminergic system in AD, which begins plateauing in the prodromal stages.	^123^ I-FP-CIT	Dopamine	HC (n = 74), MCI (n = 16) and AD (n = 22)	Pairwise correlation
Fang et al. (2021)	Identified consistent, coherent patterns of synaptic density variability in healthy individuals, suggesting that these networks contain both complementary and unique information compared to ^18^ F-FDG PET and rs-fMRI.	^11^ C-UCB-J	Synaptic Density	HC (n = 80)	ICA
DuBois et al. (2021)	Revealed abnormalities in large-scale mGluR5 networks linked to the duration of epilepsy in FCD patients.	^11^ C-ABP688	Metabotropic Glutamate Receptor Type 5	HC (n = 33) and Epilepsy (n = 17)	Jensen- Shannon divergence
Rebelo et al. (2021)	Showed that functional and molecular forms of brain plasticity are related in PD, establishing a tight link between functional activation and synaptic changes at the molecular level.	^11^ C-Raclopride	Dopaminergic D _2_ Receptors	HC (n = 9) and PD (n = 14)	Pairwise correlation
Peng et al. (2021)	Suggested that dual-phase ^18^ F-FPCIT PET is a viable methodology for quantitatively assessing PD-related metabolic brain networks and presynaptic nigrostriatal dopaminergic functioning in a single imaging session, serving as an alternative to ^18^ F-FDG PET.	^18^ F-FPCIT and ^18^ F-FDG	Dopamine Transporter and Glucose metabolism	HC (n = 16) and PD (n = 25)	SSM-PCA
Colloby et al. (2021)	Derived spatial patterns that distinguish PD from healthy individuals, correlating with global cognition, motor severity, and cognitive decline in PD patients.	^123^ I-QNB and 99mTc-exametyzime	Muscarinic Receptors M1 and M4 and Cerebral blood flow	Elderly controls (n = 24) and PD (n = 19)	PCA spatial covariance
Sanchez-Catasus et al. (2022)	In vivo evidence of the imbalance between acetylcholine and dopamine signalling systems in the striatum in early PD.	^18^ F-FEOBV and ^11^ C-DTBZ	Acetylcholine– Dopamine Transporters	HC (n = 15) and PD (n = 45)	Inter-regional correlation
Boccalini et al. (2022)	Nigrostriatal bindings and connectivity were more altered in male than female PD subjects, providing unique evidence of gender effects on the molecular connectivity of both dopaminergic systems affected by the disease.	^123^ I-FP-CIT	Dopamine	HC (n = 73) and PD (n = 286)	Pairwise correlation
Boccalini et al. (2023)	Connectivity of the nigrostriatal and mesolimbic systems was affected, with different patterns observed between males and females affected by DLB.	^123^ I-FP-CIT	Dopamine	Controls (n = 78) and pDLB (n = 123)	Pairwise correlation
Caminiti et al. (2023)	Provided the first evidence of widespread adaptive reconfigurations of dopaminergic networks across the continuum of Lewy body disease.	^123^ I-FP-CIT	Dopamine	Controls (n = 52), pDLB (n = 20) and DLB (n = 29)	Pairwise correlation
Fang et al. (2023)	Found potential links between RSN activity and ^11^ C-UCB-J source networks, indicating that synaptic density networks may be intricately connected to the functioning of large-scale intrinsic brain networks.	^11^ C-UCB-J	Synaptic Density	HC (n = 34)	ICA
Liu et al. (2023)	Identified differences in the symmetry and severity of dopaminergic dysfunction between two PD gene mutations.	^18^ F-CFT and ^18^ F-FDG	Dopamine Transporter and Glucose metabolism	PD (n = 28)	Inter-regional correlation
Smith et al. (2023)	Identified a spatial covariance pattern that distinguished MCI from healthy controls, characterised by lower serotonin transporter availability and greater cortical amyloid deposition.	^11^ C-DASB and ^11^ C-PIB	Serotonin Transporter and Amyloid burden	HC (n = 27) and MCI (n = 22)	PLS
Luo et al. (2023)	They found that STN-DBS could modify the cerebral network without preventing striatal DAT decline and confirmed its effectiveness as a therapeutic approach for controlling symptoms in patients with PD.	^18^ F-FDG and ^11^ C-CFT	Glucose Metabolism and Dopamine Transporter	PD (n = 12)	SSM-PCA
Pedersen et al. (2024)	Demonstrated that D1 receptor organisation followed a unimodal–transmodal hierarchy, with a high spatial correspondence to the principal gradient of functional connectivity.	^11^ C-SCH23390	Dopamine	HC (n = 176)	Low dimensional manifold representation
Boccalini et al. (2024)	Distinct clinical and molecular trajectories were observed in PD and SWEDD subjects.	^123^ I-FP-CIT	Dopamine	HC (n = 49), SWEED (n = 36) and idiopathic PD (n = 49)	Pairwise correlation

In terms of study populations, 34 studies included healthy control (HC) subjects, 15 focused on PD, 4 on mild cognitive impairment (MCI), 3 on Schizophrenia, 3 on Dementia with Lewy bodies (DLB), 3 on MDD, and 2 on AD. Only 1 study investigated anxiety, epilepsy, cocaine-use disorder (CUD), and attention-deficit/hyperactivity disorder (ADHD). Pairwise correlation was the most frequently applied methodology to investigate molecular connectivity (14 studies), followed by inter-regional correlation (10 studies), PCA-base approaches (6 studies), and ICA (4 studies). Partial least squares (PLS) was employed in 2 studies, while the remaining studies used combinations of these approaches, or “non-conventional” approaches usually not employed in this field.

Finally, in terms of the type of analysis conducted, 34 studies performed inter-subject analyses, where comparisons are made between different individuals or groups to identify variations across subjects. 4 studies included both inter-subject and intra-subject comparisons, the latter involving analyses within the same individual over time or under different conditions. Only 1 study focused solely on intra-subject analysis.

#### 3.2.1 Dopamine system

Several studies employed PET imaging to investigate imaging-derived dopamine-weighted networks across various neurological conditions.

Yasuno et al. (2005)applied structural equation modelling to^11^C-FLB457 brain PET imaging of HC and Schizophrenia patients. Using this method, the inter-regional correlations of D_2_receptor binding were decomposed to assign numerical weights (called path coefficients) to the anatomical connections and to evaluate the effective connectivity of regional D_2_receptor binding in Schizophrenia. The strength and signs of these path coefficients were compared between groups and used to identify disease-specific changes in the connectivity of regional D_2_receptor binding within the same anatomical networks.

Kaasinen et al. (2006)employed regional Pearson correlation and PCA to investigate corticostriatal profiles of glucose consumption and extrastriatal dopamine synthesis capacity covariance patterns in PD. The examination of striatal tracer binding revealed asymmetrical sex-dependent uptake of^18^F-FDOPA in the putamen, while revealing negative correlations between striatal^18^F-FDOPA uptake and PD clinical severity. None of these findings were seen with^18^F-FDG. Similarly, the network analysis using PCA revealed a specific component related to thalamus and cerebellum in^18^F-FDG uptake associated with both^18^F-FDOPA uptake and disease severity. On the contrary, univariate analysis showed poor correlations between^18^F-FDOPA and^18^F-FDG uptake in PD when using raw regional uptake.

Cervenka et al. (2010)examined the relationship between dopamine D_2_receptors across all brain regions in HC.^11^C-FLB457 PET was used to measure binding in extrastriatal regions, while^11^C-raclopride was employed for the measurements of D_2_distribution in the striatum. Pairwise correlations were calculated between regional*BP*_ND_values of^11^C-raclopride, and a voxel-based correlation analysis was performed using parametric images of^11^C-FLB457 binding. Additionally, correlations between regional-based*BP*_ND_values and parametric values were assessed for each region separately. The results showed that striatal receptor availability did not exhibit statistically significant correlations with any of the extrastriatal regions. These findings suggested that striatal dopaminergic biomarkers may not serve as a reliable index for global dopamine function, and results do not support using the striatum as an index for global D_2_receptor availability.

Caminiti et al. (2017)characterised presynaptic dopamine activity in early PD patients using^11^C-FeCIT PET and assessed connectivity within nigrostriatal and mesolimbic systems using partial correlation. The aim of the study was to assess—by means of univariate and multivariate approaches—if the axons of the nigrostriatal dopaminergic system are an early site for vulnerability in PD. The findings indicated greater presynaptic degeneration in dorsal putamen than substantia nigra, and more severe molecular connectivity alteration in the nigrostriatal than mesolimbic pathway.

Worhunsky et al. (2017)examined D_2_and D_3_receptors alterations in the midbrain, striatum and other subcortical structures in individuals with CUD and HC. The aim was to apply ICA on^11^C(+)-PHNO PET*BP*_ND_data with the objective of unmix the D_2_and D_3_components of*BP*_ND_and examine distinct sources of receptor availability. ICA analysis identified three distinct source-based patterns of*BP*_ND_, suggesting that cocaine-related alterations in D_2_and D_3_may not be limited to the dorsal striatum and midbrain respectively but may extend into the pallidum and ventral striatum. Furthermore, these alterations sources were associated with duration of cocaine use and may indicate reciprocal and compensatory mechanisms of dopaminergic function in addiction.

Klyuzhin et al. (2018)applied PCA to identify voxel covariance patterns, and LASSO to optimally combine several patterns. These approaches were applied to analyse dopaminergic PET tracers (^11^C-DTBZ and^11^C-raclopride) binding in the striatum of PD subjects. The principal component (PC) loadings obtained in different groups of subjects revealed predominant voxel-level binding patterns associated with the initial symptom onset and disease progression. The PC-LASSO estimators captured information in a non-local manner, and hence enabled data-driven visualisation and interpretation of spatial patterns manifested in the images.

J. Kim et al. (2018)investigated interregional correlations of D_2_and D_3_receptor availability in Schizophrenia patients receiving antipsychotics using^18^F-fallypride PET and resting state-fMRI, revealing altered molecular and functional connectivity between striatal and extrastriatal regions in stable outpatients with schizophrenia on antipsychotics, which is mainly characterised by increased interregional relationships. These results suggested that the spatial organisation of D_2_and D_3_receptor availability and related functional connectivity were significantly perturbed in these subjects.

Fu et al. (2019)introduced a joint pattern analysis approach, canonical correlation analysis and orthogonal signal correction to identify characteristic spatial and temporal distribution patterns in PD using^11^C-DTBZ (VMAT_2_marker) and^11^C-MP (DAT marker) PET data. Results showed that the proposed approach was able to capture the spatial and temporal disease patterns with higher sensitivity compared to univariate analysis. The approach provided information not only on localised alterations but also on the spatial extent of such alterations, emphasizing network behaviour of the molecular targets under investigation. Moreover, the approach decomposed the common information between data sets into distinct orthogonal patterns of characteristic dopaminergic changes that were more sensitive either to disease discrimination or to disease progression.

Veronese et al. (2019)conducted a graph-based analysis across different PET tracers (^18^F-FDG,^18^F-FDOPA,^11^C-SB217045) both in controls and in diseased groups (AD and MCI), revealing that these metrics can complement standard PET analysis to understand how biological functions are organised across brain regions in healthy and pathological conditions. The study also showcased the sensitivity of connectivity results to experimental design and variables, including group inhomogeneity and image resolution, and suggested that further methodological work is required to validate the use of more complex network metrics in the context of PET covariance analysis and to understand their biological interpretability.

Verger et al. (2020)investigated the feasibility and potential of molecular connectivity using neurotransmission tracers (^18^F-FDOPA and^123^I-FP-CIT) compared to metabolic connectivity (^18^F-FDG) in dopaminergic pathways of HC. Through interregional correlation analysis to construct a brain connectivity network, the study demonstrated that specific neurotransmission tracers provide higher specificity in revealing the mesotelencephalic system (nigro-striatal, mesolimbic, and mesocortical pathways) compared to metabolic connectivity. Notably,^18^F-FDOPA was more effective than^123^I-FP-CIT in identifying the mesotelencephalic system, indicating that these dopaminergic targets are not equivalent. The findings underscore the advantages of using^18^F-FDOPA PET imaging for molecular connectivity, highlighting its superior sensitivity and specificity relative to^18^F-FDG metabolic connectivity and emphasising that the choice of imaging modality and neurotransmitter targeting is crucial.

Smart et al. (2020)assessed the utility of four-dimensional ICA application to a competition binding PET study using^11^C(+)-PHNO PET tracer with the D_3_antagonist ABT-728, for the estimation of subtype-specific receptor occupancy. The results showed that ICA identified two distinct components of change in binding on the basis of spatiotemporally coherent variance across subjects and time points. The spatial sources of these components were highly consistent with D_2_and D_3_related^11^C(+)-PHNO binding distributions in the brain, suggesting that this analysis successfully separated each receptor subtype without any a priori assumptions. This interpretation was further supported by relative changes in the intensity of each source during blockade with the D_3_-selective antagonist ABT-728, which were closely matched to region-based occupancy estimates.

Mihaescu et al. (2021)performed a graph theory analysis of D_2_receptors measured with^11^C-FLB-457 in two brain networks: the meso-cortical dopamine network and the meso-limbic dopamine network in PD patients with cognitive decline. The findings suggested how connectivity dysregulation in extrastriatal dopamine networks may contribute to cognitive decline. Furthermore, this study wanted to highlight that multivariate network analysis captured different aspects of the dopaminergic dysfunction compared to univariate regional comparisons of localised receptor density differences.

Sala et al. (2021)examined the molecular connectivity alterations in AD, MCI, and HC subjects’ data measured with^123^I-FP-CIT SPECT tracer using partial correlation with gender, age, and reconstruction method included as nuisance covariates. The study provided biological in vivo evidence for a significant derangement of the meso-limbic dopaminergic system in AD, already plateauing in the prodromal stages. Both in vivo dopaminergic binding density and molecular connectivity analysis pointed to different degrees of vulnerability of the dopaminergic afferents from specific dopaminergic nuclei.

Rebelo et al. (2021)used covariance statistics at molecular and functional levels (measured through fMRI) to explore striato-cortical links in PD in on/off medication states using^11^C-Raclopride PET tracer. The study showed that functional and molecular forms of brain plasticity are related. These authors found a tight link between functional activation and synaptic changes at the molecular level, reflecting network reorganisation of compensatory molecular and functional mechanisms in PD.

Peng et al. (2021)found that PD-related pattern expression levels, calculated using SSM-PCA, and measured in early-phase^18^F-FPCIT PET scans, discriminated patients with early-stage PD from age-matched HC subjects with similar accuracy for the first 2, 5, and 10 min of the dynamic^18^F-FPCIT PET acquisitions. These results suggested that dual-phase^18^F-FPCIT PET is a viable methodology for quantitative assessment of PD-related metabolic brain networks, as an alternative to^18^F-FDG PET, and presynaptic nigrostriatal dopaminergic functioning in a single imaging session.

Sanchez-Catasus et al. (2022)examined the striatal acetylcholine–dopamine imbalance hypothesis in early PD patients using dual-tracer PET and dopaminergic PET–informed correlational tractography. Firstly, the authors estimated the integrity of the dopaminergic nigrostriatal white matter tracts in PD subjects by incorporating molecular information from striatal^11^C-DTBZ into the fibre-tracking process using correlational tractography (based on quantitative anisotropy (QA)). Subsequently, they used voxel-based correlation to test the association of the mean QA of the nigrostriatal tract of each cerebral hemisphere with the striatal^18^F-FEOBV*DVR*in PD subjects. The same analysis was performed for^11^C-DTBZ*DVR*in 12 striatal subregions. Taken together, results provided in vivo evidence of the imbalance between acetylcholine and dopamine signalling systems in the striatum in early PD.

Boccalini et al. (2022)investigated gender differences in the molecular connectivity of the dopaminergic systems using a large PPMI cohort of newly diagnosed and drug-naïve idiopathic PD patients measured with^123^I-FP-CIT SPECT. Partial correlation was used to assess regional co-variation in tracer uptake across subjects, and percentage of altered molecular connections in each network was used to quantify the severity of connectivity alterations between males and females. Results showed that nigrostriatal bindings and connectivity were more altered in males than females, providing unique evidence of gender effects in molecular connectivity of both dopaminergic systems affected by the disease.

Liu et al. (2023)conducted a dual-tracer PET study employing both^11^C-CFT DAT imaging and^18^F-FDG imaging to compare dopaminergic dysfunction and glucose metabolism characteristics in early-onset PD caused by different gene mutations (PRKN-EOPD and GU-EOPD) using seed-based correlation analysis. Results demonstrated differences in the symmetry and severity of dopaminergic dysfunction between the two gene mutations, suggesting potential network reorganisation due to compensatory mechanism in PRKN-EOPD which did not occur in those with GU-EOPD.

Boccalini et al. (2023)aimed to investigate molecular connectivity alterations in nigrostriatal and mesolimbic dopaminergic pathways focusing on sex differences by using^123^I-FP-CIT binding in striatal and extrastriatal regions in patients with probable DLB (pDLB). Assessment of molecular connectivity between targets of each dopaminergic pathway was performed via partial correlation analysis, and percentage of altered molecular connections in each network for males and females was calculated to quantify the severity of connectivity alterations. Results showed that connectivity of the nigrostriatal and mesolimbic systems was affected in both sex groups but with different patterns, with pDLB females showing more long-distance connectivity alterations between subcortical and cortical regions of the dopaminergic systems.

Caminiti et al. (2023)using^123^I-FP-CIT SPECT imaging adopted correlation analysis to assess the involvement of the ventral and dorsal dopaminergic circuitries in prodromal and clinical phases of DLB. Correlation analyses assessed the significant differences in connectivity between each clinical group and a subgroup of control subjects. This work provided the first evidence of widespread adaptive reconfigurations of dopaminergic networks in the continuum of Lewy body disease. The dopaminergic network showed an extensive increase of connectivity in prodromal phases, both in dorsal and ventral dopaminergic systems, supporting adaptive/compensating mechanisms, whereas a widespread loss of connectivity was prominent in overt DLB.

Luo et al. (2023)using^11^C-CFT and^18^F-FDG PET imaging investigated the effects of Subthalamic nucleus (STN) deep brain stimulation (DBS) on the distribution of presynaptic DAT and the pattern of cerebral glucose metabolism in PD patients before and after surgery. By applying SSM-PCA, they found that STN-DBS could modify the cerebral network without preventing striatal DAT decline. On the other hand, UPDRS-III scores, particularly resting tremor and rigidity, were significantly reduced after STN-DBS surgery, confirming that STN-DBS is an effective therapeutic approach in controlling symptoms in patients with PD.

Boccalini et al. (2024)investigated dopamine transporter, using semiquantitative^123^I-FP-CIT SPECT imaging, in a large cohort of idiopathic PD patients, healthy subjects and Scan Without Evidence of Dopaminergic Deficit (SWEDD) cases. Their covariance statistics analysis highlighted distinct clinical and molecular trajectories of PD and SWEDD subjects. SWEDD subjects were characterised by prominent non-motor symptoms, absence of hyposmia, and generally preserved dopaminergic binding, but prevalent mesocorticolimbic connectivity impairment, suggesting other mechanisms contributing to SWEDD pathophysiology.

Finally, by using the world’s largest combined^11^C-SCH23390 D_1_receptors PET and MRI dataset from the DyNAMiC study,Pedersen et al. (2024)tested the hypothesis that D_1_receptors organisation is aligned with functional architecture and that inter-regional relationships in D_1_receptors co-expression modulates functional cross-talk in control subjects. They applied a nonlinear embedding approach where functional and dopaminergic organisations were characterised as a set of low-dimensional manifolds and extended this analysis also to individual participants. Results demonstrated that D_1_receptors organisation followed a unimodal–transmodal hierarchy, expressing a high spatial correspondence to the principal gradient of functional connectivity. They also demonstrated that individual differences in D_1_receptors density between unimodal and transmodal regions were associated with functional differentiation of the apices in the cortical hierarchy. Finally, they showed that spatial co-expression of D_1_receptors primarily modulates couplings within, but not between, functional networks. Together, these results showed that D_1_receptors co-expression provides a biomolecular layer to the functional organisation of the brain.

#### 3.2.2 Serotonin system

Several studies have also investigated the brain network alterations in serotonin neurotransmission, mainly in neuropsychiatric disorders.

Hahn et al. (2010)explored the association of serotonin-1A receptor binding obtained with^11^C-WAY-100635 PET imaging in the dorsal raphe nucleus and the entire brain in anxiety disorder patients before and after escitalopram treatment using covariance statistics, revealing enhanced autoreceptor-to-heteroreceptor binding correlation after treatment. Results underlined the evaluation of neurotransmitter systems on a network level potentially provides important complementary information to regional receptor levels.

Tuominen et al. (2014)applied a seed-based voxel-wise correlation analysis method for studying internal neurotransmitter network structure and intercorrelations of different neurotransmitter systems in the human brain of HC subjects. They evaluated serotonin transporter (^11^C-MADAM) and µ-opioid (^11^C-Carfentanil) receptor*BP*_ND_intra- and intercorrelations. The analyses revealed nonuniformity in the serotonin transporter intracorrelations and identified a highly connected local network. Regionally specific intercorrelations between the opioid and serotonin tracers were found in areas relevant to several neuropsychiatric disorders, especially affective disorders.

Hahn et al. (2014)investigated serotonin transporter associations using^11^C-DASB PET tracer in major depression from a network perspective, revealing disturbances in a major serotonin pathway. They identified the disturbance of a major 5-HT pathway in MDD through an interregional correlation approach. Results suggested a reduced serotonin transporter association between the midbrain dorsal raphe and the ventral striatum/nucleus accumbens complementing the biological mechanisms of anhedonia in major depression and further underline the importance of the serotonergic system in reward processing. These results emphasised the importance of investigating neurotransmitter systems on a network level.

Nørgaard et al. (2017)employed a multivariate PLS approach to identify a pattern of serotonin transporter (5-HTT) levels, measured with^11^C-DASB PET imaging, fluctuating with group and season in seasonal affective disorder (SAD) a subtype of MDD. The method was able to identify and map a whole-brain pattern of 5-HTT levels that distinguished the brains of females without SAD from females suffering from SAD.

Vanicek et al. (2017)investigated the altered interregional molecular associations of the serotonin transporter in ADHD using PET imaging. They utilised^11^C-DASB PET to assess SERT binding potential in regions rich in SERT and observed differences in SERT availability between adult patients with ADHD and healthy controls. Additionally, they conducted a correlational analysis to examine the interregional association of SERT binding, finding significant interregional differences in SERT*BP*_ND_correlations.

Fu et al. (2018)applying SSM-PCA to^11^C-DASB PET data, identified a serotonergic spatial covariance pattern characteristic of PD, strongly correlated with disease duration and dopaminergic denervation measured with^11^C-DTBZ PET imaging. The study highlighted that compared to previously used univariate analysis approaches, the spatial covariance method was found to be more sensitive in identifying disease-related abnormalities since no correlation between DTBZ and DASB*BP*_ND_values of individual regions was found, suggesting PD affects the serotonergic system on a more global network level rather than any particular region in isolation. These findings suggested that disease-induced alterations of the serotonergic system, rather than being purely local, also affect interactions between separate regions in a disease-specific fashion and are closely linked to abnormalities in the dopaminergic system.

Similarly,Pillai et al. (2019)investigated molecular connectivity disruptions in MDD using covariance statistics applied to^11^C-WAY-100635 PET data. Results showed compromised structural and compensatory mechanisms of post-synaptic receptor regulation in MDD men. Interestingly, the study suggested that these individual differences in molecular connectivity between HC and MDD were so large that they may serve as a biomarker for the disorder.

Fazio et al. (2020)examined the impairment of serotonin transporter availability measured with^11^C-MADAM PET in early non-depressed PD patients using covariance statistics and graph metrics, detecting network changes preceding overt depletion in the serotoninergic system. The findings indicated that the serotoninergic system might become involved in PD patients as the disease progresses and importantly this finding was only captured by network measures, but not by direct regional binding.

Smith et al. (2023)studied the association between serotonin degeneration measured with^11^C -DASB and beta-amyloid deposition in mild cognitive impairment measured with^11^C-PIB using a multi-modal PLS algorithm. This approach identified a spatial covariance pattern that distinguished MCI from healthy controls characterised by lower serotonin transporter availability and greater cortical amyloid deposition. The pattern was expressed to a significantly greater extent in the MCI relative to the control group and was correlated with impairment in memory and executive function in the MCI group.

#### 3.2.3 Opioid system

Only two studies employed covariance analysis to investigate the modulation of µ-opioid receptor activity and its implications in disease conditions.Wager et al. (2007)investigated the placebo effects on µ-opioid receptor binding potential using^11^C-carfentanil PET imaging in HC. Through interregional correlations, the authors found that placebo treatment increased functional connectivity between µ-opioid-rich limbic and paralimbic regions, suggesting a mechanism for placebo-induced pain relief mediated by the endogenous opioid system.

In contrast,Ashok et al. (2019)utilised^11^C-carfentanil PET imaging to examine µ-opioid receptor availability in Schizophrenia patients. Their findings revealed reduced µ-opioid receptor availability in the striatum and brain regions associated with hedonic responses compared to healthy controls. Furthermore, correlation analysis indicated a significant global increase in µ-opioid receptor connection strength in Schizophrenia patients relative to controls, highlighting aberrant µ-opioid system activity in the context of Schizophrenia pathology.

#### 3.2.4 Muscarinic receptor system

Colloby et al. (2020)conducted two studies investigating cholinergic muscarinic M1/M4 receptor networks in DLB and PD, respectively. In the study on DLB, they utilised spatial covariance analysis on^123^I-QNB SPECT scans to explore muscarinic M1/M4 connectivity in Cholinesterase Inhibitor (ChEI) naive patients. They identified baseline spatial covariance patterns of M1/M4 receptors that distinguished DLB from healthy individuals and were associated with positive changes in global cognition and neuropsychiatric symptoms after ChEI treatment. These findings suggested that specific brain regions play a crucial role in the neuropsychiatric profile of DLB. In the PD study (Colloby et al., 2021), they employed a similar approach using^123^I-QNB SPECT scans to derive patterns distinguishing PD from healthy individuals and correlating with global cognition, motor severity, and cognitive decline in PD patients. They identified multiple cholinergic muscarinic receptor networks in PD, with cognition and motor severity showing similar topography, suggesting related cholinergic mechanisms underlying both phenotypes. The relative decrease in M1/M4 receptor expression within default mode network and frontal executive hubs could potentially serve as an indicator of future cognitive decline in PD.

#### 3.2.5 Glutamate receptor system

DuBois et al. (2021)conducted a study aiming to characterise the mGluR5 network in patients with focal cortical dysplasia (FCD) using^11^C-ABP688 PET imaging. Through graph theoretical analysis based on the comparison of probability density function of each regional*BP*_ND_, at the individual subject level, calculated by Jensen-Shannon divergence, they revealed abnormalities in large-scale mGluR5 networks linked to the duration of epilepsy in FCD patients. Their findings indicated decreased resilience and global efficiency, suggesting a less integrated network in FCD patients. These results support the notion that FCD may be better understood as a system-wide disorder rather than a focal abnormality from a glutamatergic neuroreceptor perspective. The graph approach employed in this study allows for the comparison of neuroreceptor systems imaged with PET to other measures of functional and structural connectivity, offering insights into the broader neurological implications of FCD.

#### 3.2.6 Synaptic density

The studies byFang et al. (2021)delve into the exploration of synaptic density networks. In the first study, the authors employed ICA on^11^C-UCB-J PET data to identify coherent patterns of synaptic density variability in healthy individuals. The analysis revealed sample-independent networks consistently extracted across different model orders, suggesting that these networks contain both complementary and unique information compared to^18^F-FDG PET and resting state-fMRI. In their second study,Fang et al. (2023)expanded on this by using ICA to examine associations between resting-state network (RSN) fluctuations and synaptic density using multimodal fMRI and^11^C-UCB-J PET in healthy controls. They found potential links between RSN activity and^11^C-UCB-J source networks, indicating that synaptic density networks may be intricately connected to the functioning of large-scale intrinsic brain networks. These findings shed light on the relationship between synaptic physiology and brain network organisation that can be captured through the application of connectivity/multivariate approaches, providing valuable insights into the underlying mechanisms of brain function.

## 4 Discussion

In this paper, we explored the use of molecular imaging to study neurotransmission through a connectivity lens. We reviewed studies involving healthy volunteers, neurological diseases, psychiatric disorders, and other conditions, aiming to encompass all relevant applications discussed in the literature. We examined studies employing various methods, such as covariance statistics and network analyses, which complement traditional univariate approaches. Collectively, these findings reveal patterns of molecular connectivity that are essential for understanding disease mechanisms.

### 4.1 The value of molecular connectivity for studying neurotransmission

The reviewed studies highlight how molecular connectivity is frequently employed alongside traditional univariate analyses to strengthen research findings. Often, multivariate and network-based methods are used to complement and validate results from conventional univariate approaches, enhancing the robustness and reproducibility of conclusions by offering a broader contextual perspective and reinforcing initial insights approaches (Ashok et al., 2019;Boccalini et al., 2024;Caminiti et al., 2017;Hahn et al., 2010;Sala et al., 2021;Vanicek et al., 2017;Wager et al., 2007). Beyond simply confirming results, molecular connectivity has also provided novel insights and uncovered patterns otherwise undetectable through univariate methods alone (Boccalini et al., 2024;Colloby et al., 2020;Kaasinen et al., 2006;Liu et al., 2023;Smart et al., 2020;Worhunsky et al., 2017). This integrated approach is capable of merging localised insights with broader network-level relationships, increasing the robustness of findings and opening avenues for further applications.

Notably, several studies have positioned molecular connectivity as the primary analytical method (DuBois et al., 2021;Fazio et al., 2020;Fu et al., 2019;Mihaescu et al., 2021;Pedersen et al., 2024;Sanchez-Catasus et al., 2022;Veronese et al., 2019). Although this is less common, it highlights a growing focus on network-level hypotheses in PET research, where molecular connectivity serves as the main investigative tool rather than secondary support. This shift underscores the view that the understanding of brain physiology and disease mechanisms requires a global, interconnected perspective beyond isolated regional analyses (Fazio et al., 2020;Hahn et al., 2010;Sporns et al., 2005).

The literature reviewed highlights that the primary advantage of multivariate and network-based approaches is their capacity to assess molecular interactions at a systemic level. A particularly notable observation was the involvement of the dopaminergic system at a broader level, extending beyond the dopaminergic regions typically targeted by radiotracers (e.g., extrastriatal regions).

These methods reveal coordinated, disease-related changes across multiple regions and modular network alterations that affect overall system function, underscoring the necessity of a comprehensive approach to detect such patterns. Through these methodologies, researchers have identified spatial patterns of alterations and pathway disruptions linked to disease origins, reinforcing the value of network-level analysis in understanding disease aetiology (Fu et al., 2018;Kaasinen et al., 2006;J. Kim et al., 2018;Liu et al., 2023;Yasuno et al., 2005).

Similarly, “disease-specific brain networks” have been associated with stage-dependent disease changes and compensatory processes (Fu et al., 2019;Klyuzhin et al., 2018;Rebelo et al., 2021;Worhunsky et al., 2017). Such approaches effectively distinguish healthy from diseased individuals and differentiate among disease subtypes (Boccalini et al., 2022;Caminiti et al., 2023;Liu et al., 2023). Furthermore, they reveal statistically significant correlations with disease duration and cognitive measures, underscoring their clinical relevance (Colloby et al., 2020,^2021^;Smith et al., 2023).

Molecular connectivity has proven particularly insightful in studying neurodegenerative diseases, where widespread and progressive pathology is better characterised by group-level molecular covariance, as opposed to focal pathologies where individual-level analysis is required (Vallini et al., 2024). PD is the most extensively studied condition, with researchers systematically uncovering multivariate and network-level dysregulation patterns within dopaminergic pathways (Boccalini et al., 2024;Caminiti et al., 2017) and their associations with symptom onset and disease progression (Fu et al., 2019;Klyuzhin et al., 2018). Further research suggested that as PD advances, the serotonergic system may become involved, likely exhibiting a broader impact compared to the localised effects typically observed within the dopaminergic system (Fazio et al., 2020). This finding implies that the long-recognised association between dopamine and PD may not be as robust as that between serotonin and PD. Supporting this,Fu et al. (2018)reported that disease-induced alterations in the serotonergic system affect interactions between distinct brain regions in a manner specific to PD, closely tied to abnormalities within dopaminergic pathways. Ultimately, cognitive decline in PD has been linked to changes in extrastriatal dopaminergic patterns (Mihaescu et al., 2021).

Several studies have also investigated DLB, revealing notable findings.Boccalini et al. (2023)observed affected connectivity within both the nigrostriatal and mesolimbic systems in DLB, with notable sex-based connectivity variations.Caminiti et al. (2023)identified a marked increase in dopaminergic connectivity during DLB’s prodromal stages, suggesting adaptive mechanisms at work. Additionally,Colloby et al. (2020)found spatial covariance patterns in M1/M4 receptors that distinguish DLB from healthy individuals and are associated with cognitive and neuropsychiatric improvements following ChEI.

A portion of reviewed studies have explored network patterns across different radiotracers, offering insights into complementary and regulatory neurotransmitter dynamics that may contribute to specific disorders. For example,Sanchez-Catasus et al. (2022)demonstrated an imbalance between acetylcholine and dopamine signalling in the striatum during early PD.Tuominen et al. (2014)noted significant opioid-serotonin tracer intercorrelations in regions linked to neuropsychiatric conditions, especially affective disorders, whileSmith et al. (2023)distinguished MCI from healthy controls through a serotonin and amyloid covariance pattern.

Notable findings have also emerged from studies comparing or integrating molecular connectivity with other imaging modalities.J. Kim et al. (2018)noted spatial alterations in D_2_and D_3_receptor availability post-antipsychotic treatment, aligning with functional connectivity changes, whileRebelo et al. (2021)demonstrated connections between functional activation and molecular-level synaptic changes in PD, suggesting compensatory reorganisation mechanisms.Pedersen et al. (2024)mapped D_1_receptor organisation along a unimodal-transmodal gradient closely aligned with the brain’s primary functional gradient.Fang et al. (2023)reported potential ties between resting-state network activity and synaptic density, suggesting that synaptic density may significantly influence large-scale brain network function.

Major neurotransmitter systems—including dopaminergic, serotonergic, and opioidergic systems—demonstrate strong alignment with structural and functional connectivity patterns in the brain (Hansen et al., 2022). Consequently, mapping the brain-wide distribution of radiotracers provides valuable insights into the connectivity dynamics of specific neurotransmitter systems (Hahn et al., 2019), paving the way for future multi-tracer and multi-modality studies in molecular connectivity research.

### 4.2 Methodological advances and considerations

The reviewed papers highlight several innovative methodologies for studying neurotransmission through molecular connectivity, extending beyond conventional intercorrelation techniques and covariance statistics. For instance, the method proposed byFu et al. (2019)employed joint pattern analysis canonical correlation to integrate information from multiple PET tracers allowing the evaluation of the interplay between two different receptor systems. This method demonstrated the potential of such approaches to integrate different tracers and go beyond the investigation of one receptor system at a time, which is often challenging with traditional correlation analyses. Furthermore,Sanchez-Catasus et al. (2022)proposed a dopaminergic PET-Informed Correlational Tractography which integrated the information of striatal dopamine into the white matter fibre reconstruction process to optimally guide tract reconstruction with dopaminergic specificity. The resulting metrics were then correlated to striatal dopamine and cholinergic signals, showing stronger and more robust associations with respect to non-informed tractography. Through this approach, this study showed that merging information from multiple modalities allows obtaining results with higher biological specificity.

Similarly,Worhunsky et al. (2017)applied ICA to^11^C(+)-PHNO PET*BP*_ND_data to separate D_2_and D_3_receptors distribution, effectively isolating distinct sources of radiotracer signal. This approach enabled a nuanced analysis of receptor subtypes, previously unattainable through conventional D_2_/D_3_radioligand analyses, underscoring the advantage of^11^C(+)-PHNO PET in investigating concurrent changes in D_2_and D_3_receptors.

Finally,Pedersen et al. (2024)utilised a nonlinear embedding approach to decompose group representative covariance maps into low-dimensional manifolds, elucidating transitions in covariance patterns of D_1_receptors across the cortical surface and linking dopamine expression to functional brain organisation. The proposed approach could be a valuable tool to decompose and integrate the information provided by different imaging modalities or different molecular targets, allowing to uncover possible interactions that would be difficult to establish with “common” connectivity approaches.

These methodological advancements deepen the understanding of regional relationships and facilitate the integration of molecular data among different tracers and complementary imaging modalities. This integration provides a holistic understanding of connectivity across multiple hierarchical levels and paves the way for numerous future applications.

Overall, these findings underscore the advantages of network-level approaches over traditional quantitative analyses. They offer novel insights not discernible through conventional methods, frequently extending the results obtained with univariate approaches, and provide a robust framework for integrating multiple sources of information, thereby broadening the potential scope of future applications. Nonetheless, the adoption of such techniques should be contingent upon rigorous statistical and mechanistic validation, ensuring that analyses are as complex as necessary to achieve reliable outcomes, but not unduly so.

### 4.3 Limitations and future prospectives

Despite the promise of molecular connectivity, the field faces several challenges. There is a lack of standard terminology and consistent methods, making it difficult to compare results across studies. For instance, not all studies used the terms “connectivity,” “network,” or “covariance” consistently when employing these approaches. This variability in nomenclature is also influenced by the studies’ year of publication, since throughout the years the definition of brain connectivity has changed. Only recently, the terminology used in functional connectivity with fMRI has become aligned to the PET field (Seeley et al., 2009;Volpi et al., 2023).

Another limitation is the methodological fragmentation across studies, which hinders direct comparisons between methodologies and complicates the replication and validation of results. There is a notable lack of best practices for data processing, network construction, and network statistics, as well as inadequate validation of key factors such as statistical thresholds, appropriate sample sizes, and the inclusion of normal control groups for comparison. As a result, variations in methodological approaches, including the radiotracer/target chosen, the construction of the connectivity matrix, data acquisition, scanner type and resolution, radiotracer specificity to neurotransmitter systems, off-target binding/kinetic parameter outcome of interest, processing techniques, and standardisation protocols, can lead to discrepancies in results across studies (Veronese et al., 2019).

Furthermore, the absence of robust validation mechanisms for the obtained results exacerbates these challenges. While statistical validation is common, the lack of validation through pharmacological agents or ex vivo data undermines the credibility and interpretability of the findings. One example is represented by the use of several dopamine-based PET imaging to explore cortical connections. While it is possible to investigate dopamine extrastriatal signals (see, e.g.,Olsson et al., 1999), there are brain regions where the PET signal for dopamine-target tracers is driven by perfusion and non-specific binding rather than dopamine. Therefore, even if a strong statistical effect can be found for these regions, the interpretation via dopamine-mediated mechanisms remains questionable and warrants further investigations. Additionally, the scarcity of longitudinal studies prevents the assessment of these molecular connectivity approaches in tracking network alterations and changes over time throughout the course of a disease.

Finally, most molecular connectivity results are based on group-level analyses due to the inherently “static” nature of PET images (Yakushev et al., 2017), which contain either tracer uptake values averaged over a certain time window or parametric values derived from the tracer’s dynamics. This limitation makes within-subject “fMRI-like” analysis of PET images challenging, resulting in molecular connectivity analysis typically being performed at the group level. Recently, new studies have conducted single-subject level analyses using static PET data through specific methodologies like Jensen-Shannon entropy (W. Li et al., 2023), Kullback-Liebler divergence (Wang et al., 2020), and perturbation approaches (Sun et al., 2022). However, these applications are still limited and not validated. The SSM-PCA method (Spetsieris et al., 2013) represents a validated candidate methodology for the identification of specific disease-related patterns and prospective quantification at the single-subject level. This approach assigns a score to quantify the extent to which a given patient expresses a certain disease pattern. The technique has been primarily applied to^18^F-FDG PET scans for differential diagnosis, prognosis, and tracking disease progression (Perovnik et al., 2023). However, incorporating neurotransmitter radiotracer imaging could provide deeper insights into the pathophysiology of specific diseases as demonstrated byFu et al. (2018),Luo et al. (2023), andPeng et al. (2021). Another line of research applies network-based approaches to dynamic bolus injection and infusion data, often called functional PET (fPET), attempting to mimic the approaches used for functional connectivity in fMRI, opening new possibilities for assessing molecular connectivity at the individual level. Until now, this has only been applied to^18^F-FDG PET data (Hahn et al., 2024;Jamadar et al., 2021). The only study in the literature using fPET data with a neurotransmitter tracer (^18^F-DOPA) is the one byHahn et al. (2021), although it employed only univariate analysis methods. Future studies could extend these results by applying connectivity-based approaches.

The existing literature predominantly focuses on the dopamine and serotonin systems, as these neurotransmitters are among the most frequently studied and utilised tracers. Consequently, there is a notable gap in research concerning other tracers. Further investigation into these less-explored tracers is necessary to enhance our understanding of their complementary roles in neural connectivity and to elucidate how their inclusion may advance the field’s comprehension of behaviour and disease states. Overall, molecular connectivity metrics of neurotransmission have demonstrated significant potential as biomarkers for diagnostic (e.g.,Peng et al., 2021) and prognostic (e.g.,Fu et al., 2018) purposes, and for evaluation of the effects of candidate treatments on brain function (e.g.,Colloby et al., 2020;Luo et al., 2023). To capitalise on this potential, future research should prioritise the development and application of methodologies tailored to single-subject-level analyses. Such approaches will enable more precise inferences across various measures by uncovering associations with other imaging parameters, clinical symptoms, neuropsychological deficits, and the outcomes of emerging treatments. This will foster a more personalised and accurate understanding of individual variability, enhancing the granularity of findings and their applicability to diverse populations.

Ensuring replicability and robustness of results is equally essential and can be achieved by leveraging large, diverse cohorts that include both healthy individuals and patient groups. Moreover, research should focus on evaluating changes in molecular connectivity induced by treatments and therapeutic drugs, assessing their impact across different populations.

Future longitudinal studies are particularly critical for tracking specific diseases over time. These studies will provide deeper insights into how molecular connectivity evolves and how it is influenced by disease progression or treatment outcomes. Robust biological validation should also remain a priority, with a focus on preclinical models that enable controlled experimentation. These models, including those replicating disease-specific conditions or pharmacological interventions, provide a valuable framework for testing causal relationships between biological mechanisms, molecular connectivity, and network metrics.

This integrated approach will not only advance the field of molecular connectivity but also aid in the development of personalised, effective interventions that address the unique needs of individual patients.

Ultimately, the technological advancements currently occurring in the field of molecular imaging may offer innovative solutions for validating molecular connectivity methodologies. For example, the higher sensitivity and unique spatial and temporal resolution of the new generation digital PET/CT systems, such as NeuroEXPLORER (H. Li et al., 2024), allow for imaging neurotransmitter release and small brain structures in ways that were never possible before. This enables mechanistic experiments capable of proving causality for what are now merely statistical associations. Similarly, the use of total-body long-axial-field of view scanners, such as uEXPLORER (Spencer et al., 2021), has introduced exciting possibilities to investigate the brain-body interactions (Nummenmaa, 2022). In this regard, molecular connectivity frameworks are particularly appealing statistical tools to investigate human physiology with a holistic approach. This applies not only to metabolism and inflammatory targets but also to synaptic transmission, as many body organs (i.e., gut and pancreas) contain receptor systems similar to those expressed in the brain (Bini et al., 2020).

## 5 Conclusions

In summary, this review has explored how connectivity-based methods are changing the way we study neurotransmission using molecular imaging. Our comprehensive search highlighted the diverse applications and methodologies employed in this field, with a strong emphasis on their utility in studying neurodegenerative diseases and psychiatric disorders. The integration of approaches like covariance statistics and network analyses, alongside traditional methods, has helped us gain a better understanding of how neurotransmission systems work. These methods not only reinforce results from conventional analyses but also reveal new patterns of molecular activity that are critical for understanding disease mechanisms. However, despite the promising potential of these molecular connectivity methodologies, the field still faces several challenges. The lack of consistent nomenclature, varied methods, and a lack of strong validation highlight the need to establish best practices, robust validation protocols, and validation through application in drug studies, to push this research forward and bring it into clinical use.

In conclusion, molecular connectivity research offers significant advantages over traditional methods, providing deeper insights into brain function and disease mechanisms. As the field continues to evolve, embracing these advanced methodologies will be essential to understand the complexities of the human brain and improve the robustness and applicability of research findings in clinical settings.

## Data Availability

No new data were generated or analysed in support of this research.

## Annex 1

*Search Strategy for MEDLINE/Ovid*

1. Exp Positron Emission Tomography/
2. (PET adj1 (imaging* OR scan* OR CT)).ti,ab,kf.
3. (positron adj1 emission adj1 tomograph*).ti,ab,kf.
4. Tomography, Emission-Computed, Single-Photon/
5. Single Photon Emission Computed Tomography/
6. (single-photon adj1 emission adj3 (tomograph* OR scan* OR imaging*)).ti,ab,kf.
7. SPECT.ti,ab,kf.
8. 1 OR 2 OR 3 OR 4 OR 5 OR 6 OR 7
9. Connectome/
10. connectivity OR connectome* OR connectomic* OR intercorrelation* OR covariance*.ti, ab,kf.
11. 9 OR 10
12. 8 AND 11
13. exp “Animals”/ not “Humans”/
14. 12 not 13

*Search Strategy for EMBASE*

1. positron emission tomography’/de
2. (PET NEAR/1 (imaging* OR scan* OR CT)):ti,ab,kw
3. (positron NEAR/1 emission NEAR/1 tomograph*):ti,ab,kw
4. ‘positron emission tomography-computed tomography’/exp
5. ‘single photon emission computed tomography’/exp
6. (single-photon NEAR/1 emission NEAR/3 (tomograph* OR scan* OR imaging*)):ti,ab,kw
7. SPECT:ti,ab,kw
8. #1 OR #2 OR #3 OR #4 OR #5 OR #6 OR #7
9. connectome/de
10. connectivity OR connectome* OR connectomic* OR intercorrelation* OR covariance*:ti, ab,kf.
11. #9 OR #10
12. #8 AND #11
13. ‘animal model’/exp OR ‘animal’/exp OR ‘animal experiment’/exp not Human/exp
14. #12 NOT #13

*Search Strategy for Scopus*

1. TITLE-ABS-KEY(PET W/1 (imaging OR scan OR CT))
2. TITLE-ABS-KEY(positron W/1 emission W/1 tomograph*)
3. TITLE-ABS-KEY(single-photon W/1 emission W/3 (tomograph* OR scan OR imaging))
4. TITLE-ABS-KEY(SPECT)
5. #1 OR #2 OR #3 OR #4
6. TITLE-ABS-KEY (connectivity OR connectome OR connectomic OR intercorrelation OR covariance)
7. #5 AND #6
8. INDEXTERMS (animals OR animal) AND NOT INDEXTERMS (human OR humans)
9. #7 NOT #8
